# The introduction of the fungal d-galacturonate pathway enables the consumption of d-galacturonic acid by *Saccharomyces cerevisiae*

**DOI:** 10.1186/s12934-016-0544-1

**Published:** 2016-08-18

**Authors:** Alessandra Biz, Maura Harumi Sugai-Guérios, Joosu Kuivanen, Hannu Maaheimo, Nadia Krieger, David Alexander Mitchell, Peter Richard

**Affiliations:** 1 Departamento de Bioquímica e Biologia Molecular, Universidade Federal do Paraná, Cx. P. 19046 Centro Politécnico, Curitiba, PR 81531-980 Brazil; 2 Departamento de Engenharia Química e Engenharia de Alimentos, Universidade Federal de Santa Catarina, Cx. P. 476 Campus Reitor João David Ferreira Lima, Florianópolis, SC 88040-970 Brazil; 3 VTT Technical Research Centre of Finland Ltd, P.O. Box 1000, 02044 VTT Espoo, Finland; 4 Departamento de Química, Universidade Federal do Paraná, Cx. P. 19081 Centro Politécnico, Curitiba, PR 81531-980 Brazil

**Keywords:** Ethanol, d-galacturonic acid, Saccharomyces cerevisiae, Citrus pulp, Metabolic engineering

## Abstract

**Background:**

Pectin-rich wastes, such as citrus pulp and sugar beet pulp, are produced in considerable amounts by the juice and sugar industry and could be used as raw materials for biorefineries. One possible process in such biorefineries is the hydrolysis of these wastes and the subsequent production of ethanol. However, the ethanol-producing organism of choice, *Saccharomyces cerevisiae*, is not able to catabolize d-galacturonic acid, which represents a considerable amount of the sugars in the hydrolysate, namely, 18 % (w/w) from citrus pulp and 16 % (w/w) sugar beet pulp.

**Results:**

In the current work, we describe the construction of a strain of *S. cerevisiae* in which the five genes of the fungal reductive pathway for d-galacturonic acid catabolism were integrated into the yeast chromosomes: *gaa*A, *gaa*C and *gaa*D from *Aspergillus niger* and *lgd*1 from *Trichoderma reesei,* and the recently described d-galacturonic acid transporter protein, *gat*1, from *Neurospora crassa*. This strain metabolized d-galacturonic acid in a medium containing d-fructose as co-substrate.

**Conclusion:**

This work is the first demonstration of the expression of a functional heterologous pathway for d-galacturonic acid catabolism in *Saccharomyces cerevisiae*. It is a preliminary step for engineering a yeast strain for the fermentation of pectin-rich substrates to ethanol.

**Electronic supplementary material:**

The online version of this article (doi:10.1186/s12934-016-0544-1) contains supplementary material, which is available to authorized users.

## Background

Citrus pulp and sugar beet pulp are pectin-rich wastes that are produced in considerable amounts in various countries. Citrus pulp results from the production of orange juice concentrate. Its production is concentrated mainly in Brazil and the USA, which have a share of 54 and 26 % of the global market, respectively [1]. In the 2014/2015 harvest, 1.4 million tons of orange juice concentrate (65° Brix) were produced in these two countries [1], and this amount would result in the production of about 1.9 million metric tons of citrus pulp (dry matter). Likewise, large amounts of sugar beet pulp are generated during the extraction of sugar from sugar beets in temperate countries, especially Russia, the USA and several European countries [2]. In the 2013 harvest, a total of 246 million metric tons of sugar beet were produced worldwide [2], resulting in 12.3 million metric tons of beet pulp (dry matter).

Citrus pulp and sugar beet pulp are usually sold for incorporation in cattle feed, but the costs of drying these wastes make this application barely profitable [3]. On the other hand, these wastes are potentially important sources of carbohydrates that can be used as raw materials in biorefineries for the production of bio-based chemicals and biofuels. In fact, there is a rising demand for ethanol, especially in Brazil and the USA, and biorefineries using citrus wastes or sugar beet pulp could meet part of this demand [4, 5]. However, hydrolysates obtained from pectin-rich wastes have high contents of d-galacturonic acid, which represents 18 % (w/w) of citrus waste hydrolysates [6] and 16 % (w/w) of sugar beet pulp hydrolysates [7]. Unfortunately, this uronic acid is not catabolized by *Saccharomyces cerevisiae*, which is the microorganism of choice for ethanol production.

Conversely, many microorganisms that are able to use d-galacturonic acid do not produce ethanol in appreciable amounts. *Escherichia coli*, for example, has the isomerase pathway for d-galacturonic acid catabolism but, due to low ethanol and inhibitor tolerance, it is not the preferred organism for ethanol production. Alternatively, ethanol production with *S. cerevisiae* is already a highly productive and robust process. This yeast is preferred for commercial scale ethanol production for several reasons, including the resistance to contaminants, bacteriophages, inhibitors and low pH [8]. It also tolerates higher osmotic pressures, enabling the use of a concentrated culture medium, and greater concentrations of ethanol. Considering this, the expression of a heterologous pathway for catabolism of d-galacturonic acid into *S. cerevisiae*, instead of engineering bacteria for ethanol production, is the preferred path for industrial ethanol production from hydrolysates containing d-galacturonic acid.

Different approaches have been used to engineer *S. cerevisiae* for this purpose. Huijes et al. [9], for instance, introduced by integration into the chromosomes the five genes of the bacterial isomerase pathway (*uxa*C, *uxa*B, *uxa*A, *kdg*K and *kdg*A), which converts d-galacturonic acid to pyruvate and glyceraldehyde-3-phosphate. Although Huijes et al. [9] demonstrated by qPCR that all genes were transcribed, only two enzymes of the pathway showed detectable activity after integration in *S. cerevisiae*. In another approach, Souffriau [10] expressed the fungal pathway for d-galacturonic acid catabolism in *S. cerevisiae* by cloning the four genes of the pathway (*gar*1, *lgd*1, *lga*1 and *gld*1, from *Trichoderma reesei*) in two plasmids with the *gap*-*DH*-*ADH*1 bidirectional promoter. In this work the activity of all the heterologous enzymes were detected in the cell lysate. Despite this, the recombinant yeast strain did not consume d-galacturonic acid.

In the present work, we extended the work of Soufriau [10] by integrating to the yeast chromosomes the four genes of the reductive fungal d-galacturonic catabolic pathway; in addition, we also integrated of the recently discovered d-galacturonic acid transporter from *Neurospora crassa*, in order to engineer a yeast that is able to use d-galacturonic acid as a carbon source. This work represents an important step in the construction of a *S. cerevisiae* strain able to produce ethanol from d-galacturonic acid.

## Results

### Pathway assembly and gene expression

The fungal pathway for catabolism of d-galacturonic acid was expressed in *Saccharomyces cerevisiae* strain CEN.PK111-61A, with the genes being selected from three different filamentous fungi, *Aspergillus niger*, *Trichoderma reesei* and *Neurospora crassa*.

As a first step, four genes encoding the catabolic d-galacturonate pathway in *A. niger* (*gaa*A, *gaa*B, *gaa*C and *gaa*D) were integrated into the yeast chromosomes, with each one being expressed under strong and constitutive yeast promoters (*PGK*1 or *TPI*1). The corresponding enzyme activities of *gaa*A, *gaa*C and *gaa*D were detected in the cell lysate. The corresponding activity of *gaa*B, namely l-galactonate dehydratase, was not detectable, even when a codon-optimized *gaa*B ORF was used. This result was unexpected since *gaa*B activity was demonstrated when expressed in a multi copy expression vector [11]. Considering this, *lgd*1, an l-galactonate dehydratase encoding gene from *T. reesei*, was used instead. The resulting strain (H4531), having the complete pathway (*gaa*A, *gaa*C, *gaa*D and *lgd*1) integrated, was then re-tested for the activity of all four enzymes. The enzymatic activities obtained from H4531 are listed in Table 1.

**Table 1:** Enzyme activities assayed from *S. cerevisiae* H4531 cell lysate

Enzyme	Gene	Spec. Act. (nkat/mg)
EC 1.1.1.365	*gaa*A	0.246
EC 4.2.1.146	*lgd*1	0.018
EC 4.1.2.B7	*gaa*C	0.274
EC 1.1.1.372	*gaa*D	1.139

During the course of the study, a d-galacturonic acid transporter (coded by *gat*1) was described in *N. crassa* [12]. Although a previous study reported that native *S. cerevisiae* is able to import d-galacturonic acid when grown at acidic pH values [13], the introduction of a transporter might improve the intake, especially at higher pH values. For this reason, g*at*1-*gfp* fusion protein gene was integrated into H4531, resulting in the strain H4535. The location of GAT1 was confirmed by fluorescence microscopy, with the green fluorescence of GFP being observed in the plasma membrane.

#### d-galacturonic acid consumption

Both engineered strains, H4531 and H4535, were cultivated for 5 days under aerobic conditions in YP medium, supplemented with 12 g L^−1^ of d-galacturonic acid. The *S. cerevisiae* strain CEN.PK113-1A, which does not have auxotrophies, was used as a control. Even after adaptive laboratory evolution, the recombinant strains, as well as the control strain grew poorly and did not consume d-galacturonic acid in this condition.

**Fig. 1:**
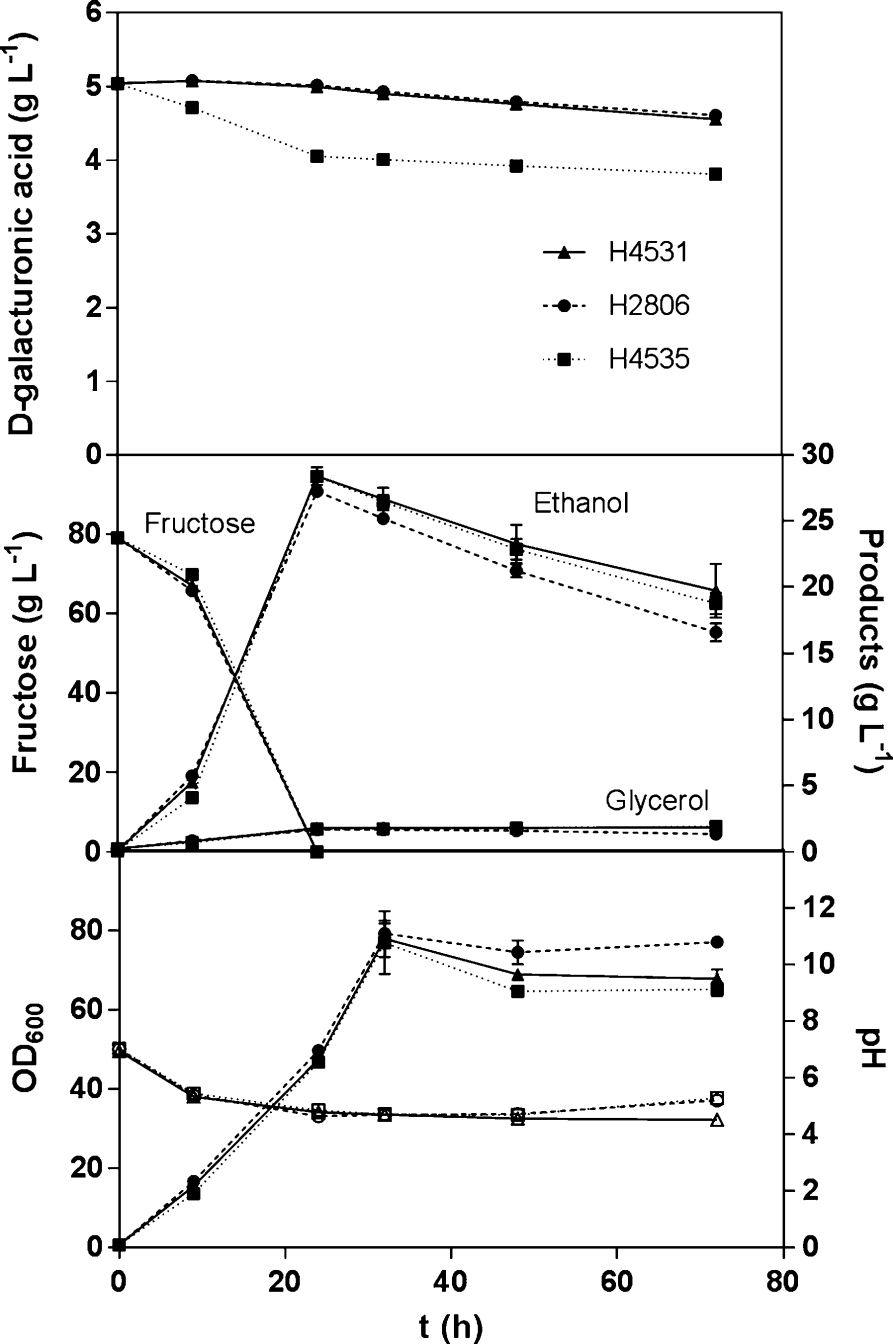
Cultivation of yeast strains in d-galacturonic acid and d-fructose. The control is H2806 (CEN.PK 113-1A), H4531 contains the genes *gaa*A, *lgd*1, *gaaC* and *gaa*D and H4535 contains the genes *gaa*A, *lgd*1, *gaa*C, *gaa*D and *gat*1. **a** d-galacturonic acid consumption. **b** Ethanol and glycerol production, fructose consumption. **c** Biomass production (*closed markers*) and pH (*open markers*). *Error bars* represent the standard error of the mean

A second fermentation was therefore done, with the difference that the medium was supplemented with 80 g L^−1^ of d-fructose (Fig. 1). This time, 20 % of the d-galacturonic acid was consumed by the yeast strain H4535, which expressed the four genes of the reductive pathway and the transporter for d-galacturonic acid. Notably, most of this consumption was observed in the first 24 h, and corresponds to the complete utilization of the d-fructose by the yeast. Consumption of d-galacturonic acid was negligible by the control strain and by the recombinant strain expressing the reductive pathway, but lacking the transporter. We could also exclude that d-galacturonic acid was simply converted to l-galactonate or other galacturonate pathway intermediates since these metabolites were not detected in neither in the culture medium nor in the cell extract. Besides the clear difference in the d-galacturonic acid consumption, every other parameter measured, such as ethanol, biomass, pH and d-fructose, did not differ significantly among the strains.

**Fig. 2:**
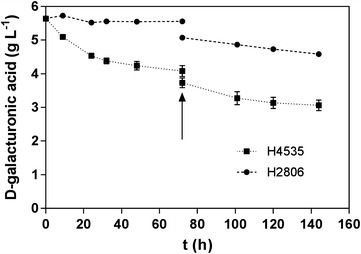
Cultivations in d-galacturonic acid and d-fructose, with a second addition of d-fructose. The control is H2806 (CEN.PK 113-1A) and H4535 contains the genes *gaa*A, *lgd*1, *gaa*C, *gaa*D and *gat*1. The fermentation was carried out with d-fructose as co-substrate, with a second load of d-fructose being added at 72 h of fermentation. d-galacturonic acid consumption was monitored over time. *Error bars* represent the standard error of the mean

To further analyse the role of d-fructose as a co-substrate that enables d-galacturonic acid consumption, an additional fermentation was performed. Here d-fructose was added in two batches of 40 g L^−1^, being the second at 72 h (Fig. 2). With this experiment was possible to observe that an additional galacturonic acid consumption followed the addition of fructose in 72 h.

**Fig. 3:**
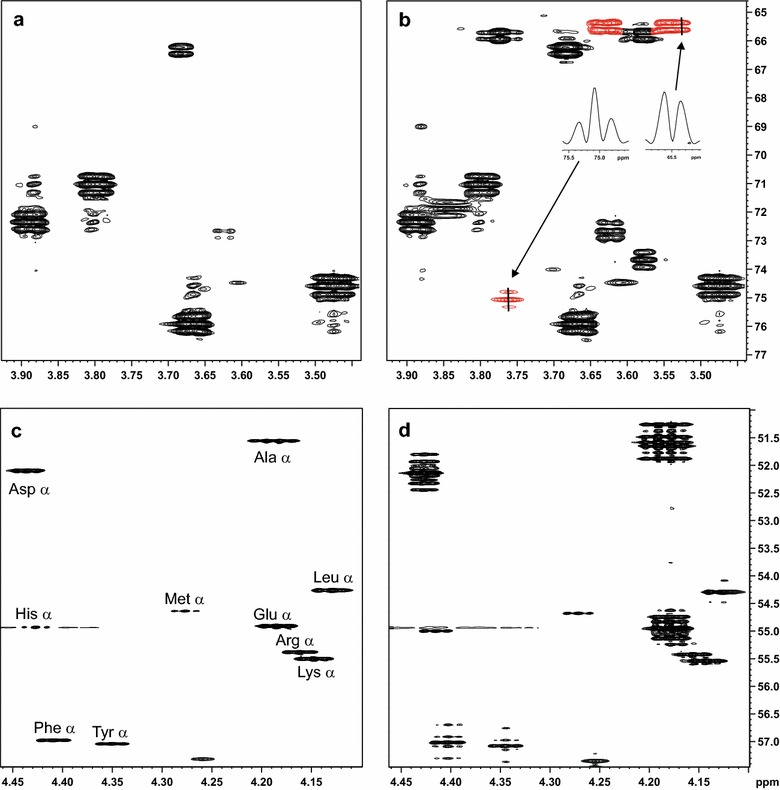
NMR analysis of the ^13^C tracing fermentations. Expansions of HSQC spectra of **a** the culture supernatant of the control strain H2806 (CEN.PK 113-1A) and **b** the strain H4535 containing the genes *gaa*A, *lgd*1, *gaa*C, *gaa*D and *gat*1. Signals of glycerol produced by H4535 are highlighted in *red* and the *inserts* show cross sections of these signals along F1, revealing the ^13^C-^13^C scalar coupling fine structure. These signals were not detected for the control strain. Part of the alpha carbon region of HSQC spectra of biomass hydrolysate of **c** the control strain and **d** the engineered strain, showing the ^13^C-^13^C scalar coupling fine structure in most signals

Finally, to prove that the galacturonic acid was definitely being catabolized by the engineered yeast, and not only converted to e.g. l-galactonate, the fate of this sugar was monitored by NMR, using the uniformly labeled d-[UL-^13^C_6_] galacturonate as substrate. For that, a fermentation was carried out with 40 g L^−1^d-fructose and 4 g L^−1^ of d-galacturonic acid, with the labeled substrate corresponding to half of the total amount of d-galacturonic acid. Again, a control strain without the pathway was fermented in the same conditions. Figure 3a, b shows the NMR spectrum for the supernatant of this fermentation at 24 h, with a corresponding to the spectrum of the supernatant from the control strain and B from the engineered strain with the fungal pathway and the transporter. In Fig. 3b, there is a clear signal of uniformly labeled glycerol showing the ^13^C-^13^C scalar coupling fine structure, meaning that the carbon backbone was originally from the labeled d-galacturonic acid. In Fig. 3a this fully labeled glycerol is not detected in the spectrum. Glycerol is a product of the d-galacturonic acid pathway and of sugar catabolism in yeast, and was detected by HPLC for both control and engineered strains in the previous experiments. The detection of uniformly ^13^C-labeled glycerol already confirms that d-galacturonic acid is metabolized by the engineered strain, and not by the control. However, to further assure that the d-galacturonic acid is being metabolized, the yeast cells were collected after 96 h of fermentation and hydrolyzed. The spectrum of the resulting amino acids was analyzed for both the control strain (Fig. 3c) and the engineered strain (Fig. 3d). Also here we observed the ^13^C-^13^C scalar coupling fine structure in the sample of the engineered strain, proving that the labeled carbon is coming from the d-galacturonic acid. The alfa carbon of several amino are ^13^C-labeled and have ^13^C-labeled neighbors in the engineered strain, but not in the control strain, which is a proof that d-galacturonic acid is catabolized and ends up in the biomass of the engineered strain.

## Discussion

In this work, we demonstrate for the first time a functional catabolic pathway for d-galacturonate consumption by *S. cerevisiae*. For this purpose, the fungal d-galacturonic acid pathway and the d-galacturonic acid transporter were integrated into the yeast chromosomes and the corresponding enzyme activities were shown in the cell lysate, and with the d-galacturonic acid transporter being located in the cell membrane. The fermentation trials with d-fructose as co-substrate showed that the engineered strain was, furthermore, able to catabolize d-galacturonic acid. The tracing of the ^13^C from uniformly labeled d-galacturonic acid showed that these carbons end up in products of the fermentation, particularly glycerol, and in the amino acids of the biomass.

**Fig. 4:**
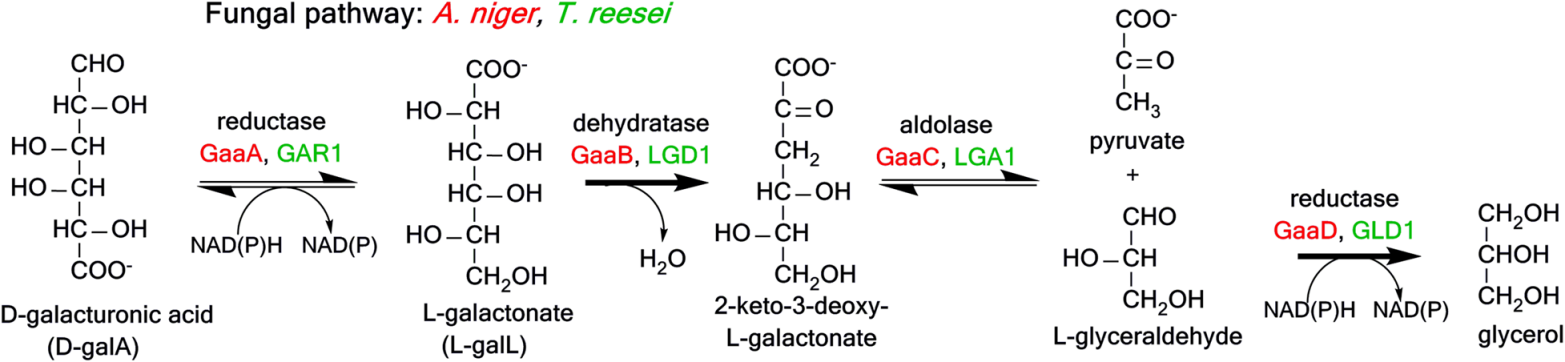
Fungal pathway for the catabolism of d-galacturonic acid

The fungal pathway for d-galacturonic acid catabolism was recently discovered by Hilditch et al. [14] and confirmed by Martens-Uznova et al. [15]. It consists of four enzymes, with two reduction steps (Fig. 4). The first enzyme of the pathway, d-galacturonate reductase (EC 1.1.1.365), coded by *gaaA* in *A. niger*, converts d-galacturonate into l-galactonate, using NADPH or NADH as the electron donor. l-galactonate dehydratase (EC 4.2.1.146), coded by *gaaB* in *A. niger* and by *lgd*1 in *T. reesei*, converts l-galactonate into 2-keto-3-deoxygalactonate. The third enzyme, 2-keto-3-deoxygalactonate aldolase (EC 4.1.2.B7), coded by *gaa*C in *A. niger*, cuts 2-keto-3-deoxygalactonate between carbons 3 and 4, producing pyruvate and l-glyceraldehyde. The pyruvate can enter the citric acid cycle, while the l-glyceraldehyde is converted to glycerol by glyceraldehyde reductase (EC 1.1.1.372, encoded by *gaa*D in *A. niger*) using NADPH as the electron donor.

Recently, Benz [12] described the d-galacturonic acid transporter, which was suggested to act as an H^+^/d-galacturonic acid symporter. The corresponding gene was discovered in *Neurospora crassa,* and to further confirm its functionality, the transporter was also co-expressed in yeast with the first enzyme of the fungal pathway, galacturonate reductase (*gaa*A).

Souffriau [10], similarly to what we have done, also expressed the genes of the fungal pathway from *Trichoderma reesei* (*gar*1, *lgd*1, *lga*1 and *gld*1) in *S. cerevisiae.* Differently from our approach, the four genes were expressed from two plasmids with the *gap*-*DH*-*ADH*1 bidirectional promoter. Despite the fact that the activity of all the enzymes was shown in the cell lysates, the recombinant yeast strain did not grow on d-galacturonic acid. Further adaptive laboratory evolution, in a medium containing both glycerol and d-galacturonic acid, did not lead to mutants that were able to use d-galacturonic acid.

Similarly to Souffriau [10], we chose the reductive pathway for introduction into *S. cerevisiae*, since previous work showed that the individual enzymes could be expressed in this yeast [8, 15–17]. However, our approach differs from that of Souffriau [10] in four ways: First, we integrated the genes into the yeast genome, instead of using plasmid vectors; second, we used genes from both *T. reesei* and *A. niger*; third, we also integrated a d-galacturonic acid transporter [12]; and, fourth, we used d-fructose, instead of glycerol, as a co-substrate for growth of the recombinant yeast. Interestingly, both Souffriau [10] and we detected the activities of the d-galacturonic acid pathway enzymes in the cell lysate, but our use of hexose sugar as a co-substrate, rather than the glycerol used by Souffriau [10] and also the use of a d-galacturonic acid transporter may be the reasons why we were able to demonstrate d-galacturonic consumption. Additionally, the use of the *A. niger*d-galacturonate reductase GAAA, which accepts both NADH and NADPH as a cofactor, instead of the *T. reesei* reductase, which strictly uses only NADPH, could also have enabled the observed d-galacturonic acid consumption.

Although the engineered strain was able to catabolize d-galacturonic acid, the strain is still not optimized for industrial processes using pectin-rich waste hydrolysates. Due to the fact that the fungal pathway requires reducing power that is also needed for ethanol production, further engineering of the strain and optimization of the cultivation conditions are still needed to address the redox balance of this process.

## Conclusion

In this work, we describe for the first time a strategy for the introduction of a functional pathway that resulted in d-galacturonic acid catabolism in *Saccharomyces cerevisiae*. This strategy was based on the integration into the yeast chromosomes of not only the four enzymes of the fungal pathway of d-galacturonic acid catabolism, but also the recently described d-galacturonic acid transporter. This represents the first step in the construction of a strain of *S. cerevisiae* that would be able to produce ethanol from d-galacturonic acid. Such a strain would find application in a citrus/sugar beet waste biorefinery, in which pectin-rich wastes would be hydrolyzed and then fermented to produce ethanol.

## Methods

### Strains

The *S. cerevisiae* strain CEN.PK111-61A was used as a parental strain for the engineered strains. The prototrophic CEN.PK113-1A was used as a control in the fermentations. All the plasmids were produced in *E. coli* TOP10 cells. Table 2 shows the original and engineered *S. cerevisiae* strains used in this work.

**Table 2:** Strains used in this work

Strains	Description	Parent strain
*Escherichia coli* TOP10	*E. coli* for electroporation *hsd*R*, mcr*A*, lac*ZΔM15*, rec*A	
CEN.PK111-61A	*S. cerevisiae* CEN.PK 111-61A MATα, *ura*3-52, *his*3-Δ1, *leu*2-3112, *TRP*1, *MAL*28^c^, *SUC*2	
CEN.PK113-1A	*S. cerevisiae* CEN.PK 113-1A MATα, *URA*3, *HIS*3, *LEU*2, *TRP*1, *MAL*28^c^, *SUC*2	
*S. cerevisiae* ATCC 90845	*MAT*α, *his*3Δ 200, *ura*3-52, *leu*2 Δ 1*, lys*2-Δ 202, *trp*1-Δ63	
*A. niger* ATCC 1015	Template DNA for gene *gaa*C	
*Schizosaccharomyces pombe*	Template DNA for gene *his*5	
H4362	*S. cerevisiae* CEN.PK 111-61A+ *gaaC* (MATα, *ura*3-52, *HIS*5, *leu*2-3112, *TRP*1, *MAL*28^c^, *SUC*2)	*S. cerevisiae* CEN.PK 111-61A
H4410	*S. cerevisiae* CEN.PK 111-61A + *gaa*C + *gaa*D (MATα, *ura*3-52, *HIS*5, *LEU*2, *TRP*1, *MAL*28^c^, *SUC*2)	H4362
H4425	*S. cerevisiae* CEN.PK111-61A + *gaa*C + *gaa*D + *gaa*A (MATα, *URA*3, *HIS*5, *LEU*2, *TRP*1, *MAL*28^c^, *SUC*2)	H4410
H4531	*S. cerevisiae* CEN.PK111-61A + *gaa*C + *gaa*D + *gaa*A + *lgd*1 (MATα, *URA*3, *HIS*5, *LEU*2, *TRP*1, *MAL*28^c^, *SUC*2*, can*1^−^)	H4425
H4535	*S. cerevisiae* CEN.PK111-61A + *gaa*C + *gaa*D + *gaa*A + *lgd*1 + *gat*1-*gfp* (MATα, *URA*3, *HIS*5, *LEU*2, *TRP*1, *MAL*28^c^, *SUC*2*, can*1^−^ *, HO* ^−^)	H4531

### Media and culture conditions

For plasmid multiplication, bacterial strains were cultivated in Luria Broth medium [18], supplemented with 100 μg mL^−1^ of ampicillin, at 37 °C and 250 rpm. For yeast transformations, several media were used. SCD: synthetic complete medium supplemented with 20 g L^−1^d-glucose [19]. SCD-URA: uracil deficient synthetic complete medium, supplemented with 20 g L^−1^d-glucose. SCD-HIS: histidine deficient synthetic complete medium, supplemented with 20 g L^−1^d-glucose. SCD-LEU: leucine deficient synthetic complete medium, supplemented with 20 g L^−1^d-glucose. SCD-URA/-HIS/-LEU: uracil, histidine and leucine deficient synthetic complete medium, supplemented with 20 g L^−1^d-glucose. YPD: 10 g L^−1^ yeast extract, 20 g L^−1^ peptone, 20 g L^−1^d-glucose [20]. YPD + G418: YPD supplemented with 200 μg mL^−1^ geneticin. YPD+ nourseothricin: YPD supplemented with 100 μg mL^−1^ nourseothricin.

### Plasmid construction and gene integrations

Plasmids are listed in Table 3. Primers used for plasmid construction are listed in Table 4. All the PCR reactions were performed using Phusion High Fidelity (Finnzymes, Finland) and Phusion High Fidelity buffer or GC Buffer (Finnzymes, Finland). For bacterial and yeast colony PCR, DyNAzyme (Finnzymes, Finland) was used instead. For yeast colony PCR, cell disruption was carried out using Zymolyase 100 T (Seikagaku Biobusiness, Japan). Ligations were done using T4 DNA ligase (Promega, USA). Recombination in yeast for plasmid assembly or genome integration was done using Lab Transformation kit (Molecular Research Reagents Inc., USA), following the protocol of Gietz et al. [21]. Recombination in vitro was done using Gibson Assembly^®^ Master Mix (New England Biolabs, USA).

**Table 3:** Plasmids used in this work

Plasmid	Description
B1181	YEplac195 with *PGK*1 promoter, *URA*3, *Amp* ^*R*^
pRS426	Yeast integration vector, *URA*3*, Amp* ^*R*^
B5430	B1181 ligated to *gaa*C. Contains fragment 3 *(PGK*1/*gaa*C*)* of the *gaa*C integration vector in between *bgl*II restriction sites
B5517	Yeast integration vector created by homologous recombination in yeast. Contains the gene *gaa*C under *PGK*1 promoter, the integration *locus HIS*3 and marker *HIS*5 (*S. pombe*) in between *Not*I restriction sites
B5470	pXY212 expressing the gene *gaa*D under *TPI*1 promoter
pRS405	Yeast integration vector, *LEU*2, *Amp* ^*R*^
B5555	Yeast integration vector pRS405 expressing *LEU*2 and *gaa*D under *TPI*1 promoter in between *Afl*II restriction sites
B2159	pXY212, containing *TPI*1 promoter region, *Amp* ^*R*^
B5706	Genescript pUC57 plasmid containing codon-optimized *gaa*A in between *Eco*RI and *Bam*HI restriction sites
B5696	B2159 ligated to *gaa*A under *TPI* promoter
pRS406	Yeast integration vector containing *URA*3, *Amp* ^*R*^
B5697	Yeast integration vector containing *URA*3 and *gaa*A under *TPI*1 promoter in between *Afl*II restriction sites
B3033	Yeast integration vector containing *CAN*1 l*ocus*, *KnM*X and *lgd*1 under *PGK*1 promoter in between *Lox*P sites and *Sac*lL and *Kpn*l restriction sites
pSH66	Deletion vector containing *Cre*, *Amp* ^*R*^
B6367	B2159 containing *gat*1-*gfp* under *TPI*1 promoter
B3531	Yeast integration vector containing *KnM*X marker, *HO* locus
B6382	B3531 yeast integration vector containing *gat*1-*gfp* under *TPI*1 promoter

**Table 4:** Primers used in this work

Primer	Sequence	Description
P1	ATGCCTTTTACCCCGCTCCG	For *gaa*C amplification from *A. niger* genome, for colony PCR and sequencing (forward)
P2	CTAAGCAATATCCGGCAACG	For *gaa*C amplification from *A. niger* genome, for colony PCR and sequencing (reverse)
P3	CGGGGGATCCACTAGTTCTAGAGCGGCCGCGTGAGGGTCAGTTATTTCAT	For fragment 1 (−1000 bp *HIS*3 locus) amplification from *S. cerevisiae* (forward)
P4	TATTTCTTTCTACAAAAGCCCTCCTACCCATCTTTGCCTTCGTTTATCTTG	For fragment 1 (−1000 bp *HIS*3 locus) amplification from *S. cerevisiae* (reverse)
P5	TAACTCGAAAATTCTGCGTTCGTTAAAGCTAGCTGCAGCATACGATATAT	For fragment 4 (+1000 bp *HIS*3 locus) amplification from *S. cerevisiae* (forward)
P6	AAGCTGGAGCTCCACCGCGGTGGCGGCCGCGGAGCCATAATGACAGCAGT	For fragment 4 (+1000 bp *HIS*3 locus) amplification from *S. cerevisiae* (reverse)
P7	AATGAGCAGGCAAGATAAACGAAGGCAAAGATGGGTAGGAGGGCTTTTGT	For fragment 2 (*his*5) amplification from *S. pombae* (forward)
P8	TTCAGTTTTGGATAGATCAGTTAGAAAGCTATTAAGGGTTCTCGAGAGCT	For fragment 2 (*his*5) amplification from *S. pombae* (forward)
P9	GGAAGATATGATCTACGTATGGTCATTTCTTC	For *TPI*1/*gaa*D amplification from B5470 (forward)
P10	GGAGATCTCGAATTGGAGCTAGAGAAAG	For *TPI*1/*gaa*D amplification from B5470 (reverse)
P11	GATCTACGTATGGTCATTTCTTC	For colony PCR and sequencing *gaa*D ORF (forward)
P12	TCGAATTGGAGCTAGACAAAG	For colony PCR and sequencing *gaa*D ORF (reverse)
P13	ATGGCTCCCCCAGCTGTGTT	For colony PCR and sequencing *gaa*A ORF (forward)
P14	CTACTTCAGCTCCCACTTTC	For colony PCR and sequencing *gaa*A ORF (reverse)
P15	CCTCGCACCCATGTACATTGG	For colony PCR and sequencing *gat*1 ORF (forward)
P16	TTATATTGGCCTTTATGTCCGC	For colony PCR and sequencing *gat*1 ORF (reverse)
P17	TATATACCCGGGGTGCCACCTGACGTCTAAGA	For amplification of *TPI1*/*gat*1-*gfp* from B6367 (forward)
P18	TATATACCCGGGAGACCGAGATAGGGTTGAGT	For amplification of *TPI1*/*gat*1-*gfp* from B6367 (reverse)

### gaaC integration

The *gaa*C integration cassette was designed to have the *gaa*C gene under the *PGK*1 promoter (fragment 3), the flanking regions of *S. cerevisiae HIS*3 gene (fragments 1 and 4) used as homologous regions for genome integration, and the *S. pombe HIS*5 gene (yeast marker, fragment 2) assembled circularly in the pRS426 vector (fragment 5), which contains a *URA*3 marker.

*gaa*C was directly amplified from *A. niger* ATCC 1015 genome. It was inserted into the *PGK*1 promoter region of B1181 plasmid using Gibson Assembly. The resulting plasmid, B5430, contains the *PGK*1/*gaa*C component (fragment 3) of the *gaa*C integration cassette and was digested with *Bgl*II to obtain fragment 3. Fragment 1, which corresponds to the-1000 bp downstream region of *S. cerevisiae his*3, and fragment 4, which corresponds to the upstream +1000 bp region of *his*3, were amplified from the *S. cerevisiae* (H3488) genome. *HIS*5 (fragment 2) was amplified from the *S. pombae* genome. The pRS426 vector was linearized by digestion with *Not*I (integration vector). The five fragments from the *gaa*C integration plasmid were assembled by recombination in yeast. The *URA*3 marker was used for the selection of the assembled plasmid and the transformants were selected in SCD-URA plates. The assembled plasmid was introduced to *E. coli* by electroporation for multiplication and digested with *Not*I for the isolation of the cassette (fragments 1 to 4). The integration of the *gaa*C cassette into the parental strain CEN.PK111-61A was done by recombination. The *HIS*5 marker was used for selection of the *gaa*C-integrated yeast and transformants were selected in SCD-HIS and confirmed by colony PCR. Activity was confirmed using the cell lysate. A colony expressing *gaa*C was stored as H4362.

### *gaa*D integration

The *gaa*D was amplified from cDNA and restricted with *Bgl*II and ligated to the B2159 plasmid in the *TPI* promoter region. *gaa*D under *TPI*1 promoter was amplified and ligated to the vector pRS405 (containing *LEU*2 yeast marker) to form B5555 integration vector. The *gaa*D integration cassette with the *LEU*2 marker was obtained by the linearization of B5555 using *Afl*II. The *gaa*D cassette was integrated into the *gaa*C-expressing yeast strain H4362, in the *leu*2-*3112* locus. Transformants were selected in SCD-LEU and confirmed by colony PCR. The activity of the *gaa*D gene was confirmed using the cell lysate. A colony expressing *gaa*C and *gaa*D was stored as H4410.

### *gaa*A integration

The *gaa*A gene was custom-synthetized as a codon-optimized ORF (Genescript). Genescript *gaa*A plasmid was digested with *Eco*RI and *Bam*HI and ligated to B2159 plasmid in the *TPI*1 promoter region. The integration plasmid B5697 was obtained by ligating the *TPI*1/*gaa*A fragment to the vector pRS406 (B704), which contains the *URA*3 yeast marker. The *gaa*A integration cassette was obtained by the linearization of B5697 using *Bsm*I. The cassette was then integrated into the *ura*3-*52* locus of the yeast strain H4410 (*gaa*C + *gaa*D). Transformants were selected in SCD-URA medium and confirmed by colony PCR. Activity was confirmed using the cell lysate. A colony expressing *gaa*C, *gaa*D and *gaa*A was stored as H4425.

### *lgd*1 integration

The integration plasmid B3013 carrying the *lgd*1 gene ready for integration in yeast was available from previous work [22]. This integration cassette consists of two flanking regions for *CAN*1, the *lgd*1 gene under the *PGK*1 promoter, and the *Kan*MX yeast marker (resistance to G418) between two *lox*P sites. The plasmid was digested with *Sac*I and *Kpn*I and the integration cassette was transformed to the yeast strain H4425 (*gaa*A + *gaa*C + *gaa*D). Before plating, the yeast cells were incubated in 2 mL of SCD for 1 h. Transformants were selected in YPD + G418 and confirmed by assaying *lgd*1 activity of the cell lysate. The *Kan*MX marker was then removed by transforming the selected yeast colony with plasmid pSH66, expressing *cre* recombinase, which removes *Kan*MX yeast marker between the *lox*P sites [23]. Before plating in YPD + nourseothricin, the yeast cells were incubated in 2 mL of SCD for 1 h. After 4 days, the resulting colonies were re-plated in YPD + nourseothricin. Biomass from this plate was inoculated into 50 mL of YP + 2 % galactose and incubated overnight. The resulting culture was plated in YPD. Some isolated colonies were replated in G418 plates and SCD-URA/-LEU/-HIS medium. After 2 days, none of the colonies grew on G418, confirming that the *Kan*MX marker had been removed. A colony expressing *gaa*C, *gaa*D, *gaa*A and *lgd*1 was stored as H4531.

### *gat*1-gfp integration

The *gat*1-*gfp* gene was custom-synthetized based on the work of Benz [12] as a codon-optimized ORF (Genescript) The *gat*1-*gfp* fragment was digested from the Genescript plasmid and ligated to B2159 plasmid, in the *TPI*1 promoter region. For the integration cassette, the *TPI*1/*gat*1-*gfp* fragment was digested with *Xma*I. The vector B3531, which contains HO integration region and *Kan*MX yeast marker, was digested with *Cfr*9I and ligated to the *TPI*1/*gat*1-*gfp* fragment. The *gat*1-*gfp* integration cassette was obtained by the digestion of the integration plasmid with *Xho*I and *Xba*I and transformed to the H4531 strain. The yeast cells were incubated in 2 mL of YPD for 2 h and then plated in YPD + G418 plates. To confirm the integration of the *gat*1-*gfp* gene, cells were incubated in SCD medium for 24 h and analysed using a fluorescence microscope. A colony showing green fluorescence (from the *gfp* reporter gene) was stored as H4535.

### Enzymatic assays

Cell extracts, obtained as follows, were used in all the enzymatic assays. Yeast cells were grown in YPD medium and collected by centrifugation, washed with water and re-suspended in phosphate buffer 50 mM, pH 7 with addition of protease inhibitor Complete EDTA Free (Roche, Switzerland). Cells were disrupted with 0.4 mm diameter glass beads using a bead beater FastPrep (MP Biomedicals, USA) and solid residues were removed by centrifugation. Protein content was assayed by the Bradford method [24].

d-Galacturonate reductase (GAAA) and l-glyceraldehyde reductase (GAAD) activities were assayed in the forward direction by following the decrease in absorbance at 340 nm caused by the oxidation of NADPH. The reaction medium for d-galacturonate reductase contained 10 mM d-galacturonic acid (Sigma-Aldrich, Germany) and 0.2 mM NADPH (Sigma-Aldrich, Germany). The reaction medium for l-glyceraldehyde reductase contained 5 mM l-glyceraldehyde (Sigma-Aldrich, Germany) and 0.2 mM NADPH (Sigma-Aldrich, Germany). The reactions of the d-galacturonate reductase and the l-glyceraldehyde reductase were followed for 5 min.

l-Galactonate dehydratase (LGD1) and 2-keto-3-deoxy-galactonate aldolase (GAAC) activities were indirectly assayed by measuring the absorbance at 549 nm, corresponding to a chromogenic compound that forms when thiobarbituric acid combines with 2-keto-3-deoxy-galacturonate [25]. The reaction medium for l-galactonate dehydratase contained 10 mM l-galactonic acid (obtained by hydrolysis of l-galactono-1,4-lactone, Sigma-Aldrich, Germany) and proceeded for 2 h. The reaction medium for 2-keto-3-deoxy-galactonate aldolase contained 10 mM l-glyceraldehyde (Sigma-Aldrich, Germany) and 10 mM pyruvate (Sigma-Aldrich, Germany) and proceeded for 30 min. l-Galactonate dehydratase was assayed in the forward direction. 2-keto-3-deoxy-galactonate aldolase was assayed in the reverse direction as its substrate, 2-keto-3-deoxy-galacturonate, is not commercially available.

### Fermentations trials

In the adaptive laboratory evolution of the transformed yeast, the medium used was YP (10 g L^−1^ yeast extract, 20 g L^−1^ peptone) pH 4,5, supplemented with 12 g L^−1^d-galacturonic acid. For assaying d-galacturonic acid consumption by transformed yeast in co-fermentation with d-fructose the medium used was YP pH 7 supplemented with 5 g L^−1^d-galacturonic acid and with 80 g L^−1^d-fructose. For evaluating the role of d-fructose in the d-galacturonic acid catabolism the medium used was initially YP pH 7 supplemented with 5 g L^−1^d-galacturonic acid and with 40 g L^−1^d-fructose. After 72 h, a concentrated d-fructose solution (40 % w/v) was added to result in the concentration of approximately 40 g L^−1^d-fructose. In the ^13^C tracing experiments, potassium d-[UL-^13^C_6_] galacturonate (Omicron Biochemicals, Inc., USA) was used for tracing the fate of d-galacturonic acid. The experiment was carried out with 2 g L^−1^ of the labeled d-galacturonic acid, 2 g L^−1^ of non-labeled d-galacturonic acid and 40 g L^−1^ of d-fructose in YP medium (neutral pH). Samples of the supernatant were taken 12 h time intervals and the biomass was collected in 96 h of fermentation. All the fermentations were carried out aerobically using 250 rpm and 30 °C. The CEN.PK 113-1A strain, which does not contain the pathway or transporter, and with similar genetic background, was used as a control in all the experiments.

### Adaptive laboratory evolution

Adaptive laboratory evolution is a technique that aims for the selection of microorganisms more adapted to grow in a certain type of medium, for example, media containing toxic compounds or non-metabolizable sugars [26]. In this work, adaptive laboratory evolution was used for the selection of mutants more suited for growing with d-galacturonic acid as a carbon source. This study was carried out with the strains H4531, and H4535, both carrying the reductive fungal pathway, with the H4535 also containing the transporter gene. The cultivation was done in YP medium pH 4.5, supplemented with 12 g L^−1^d-galacturonic acid. A dilution of 1:50 was made in fresh medium every 7 days. The cultivations continued for 8 weeks and were analysed by HPLC, as described below.

### Chemical analysis

Samples of liquid fermentation broth were taken at 12-48 h or 7-days (adaptive laboratory evolution) intervals. The concentrations of d-galacturonic acid, d-fructose and ethanol were determined by HPLC using a Fast Acid Analysis Column (100 mm × 7.8 mm, BioRad Laboratories, CA, USA) linked to an Aminex HPX-87H organic acid analysis column (300 mm × 7.8 mm, BioRad Laboratories) and 5 mM H_2_SO_4_ as eluent with the flow rate of 0.5 ml min^−1^. The column was maintained at 55 °C. Peaks were detected using a Waters 410 differential refractometer and a Waters 2487 dual wavelength UV (210 nm) detector.

### NMR analysis

The NMR experiments were carried out either at 22 °C (culture supernatants) or at 40 °C (biomass hydrolysate) on a 600 MHz Bruker Avance III NMR spectrometer equipped with a QCI cryoprobe. The culture supernatant samples were prepared by mixing 540 µl of the supernatant with 60 µl of D_2_O (Aldrich, Germany). The HCl hydrolysis of the biomass as well as the ^1^H,^13^C HSQC experiments of the aliphatic area with high ^13^C resolution are described in detail by Jouhten and Maaheimo [27]. For ^13^C decoupling during the acquisition, GARP4 was used.

## Additional file

10.1186/s12934-016-0544-1 HPLC results of the cultivations in D-galacturonic acid.
